# Spectral Radiance of a Large-Area Integrating Sphere Source

**DOI:** 10.6028/jres.100.003

**Published:** 1995

**Authors:** James H. Walker, Ambler Thompson

**Affiliations:** National Institute of Standards and Technology, Gaithersburg, MD 20899-0001

**Keywords:** calibration, integrating spheres, large-area apertures, radiometry, spatial mapping, spectral radiance, spectroradiometry

## Abstract

The radiance and irradiance calibration of large field-of-view scanning and imaging radiometers for remote sensing and surveillance applications has resulted in the development of novel calibration techniques. One of these techniques is the employment of large-area integrating sphere sources as radiance or irradiance secondary standards. To assist the National Aeronautical and Space Administration’s space based ozone measurement program, a commercially available large-area internally illuminated integrating sphere source’s spectral radiance was characterized in the wavelength region from 230 nm to 400 nm at the National Institute of Standards and Technology. Spectral radiance determinations and spatial mappings of the source indicate that carefully designed large-area integrating sphere sources can be measured with a 1 % to 2 % expanded uncertainty (two standard deviation estimate) in the near ultraviolet with spatial nonuniformities of 0.6 % or smaller across a 20 cm diameter exit aperture. A method is proposed for the calculation of the final radiance uncertainties of the source which includes the field of view of the instrument being calibrated.

## 1. Introduction

The arrival of radiometers with wide fields of view, such as instruments used in remote sensing and surveillance applications, has necessitated the development of uniform large-area radiance sources to calibrate these devices. Historically, National Institute of Standards and Technology traceable irradiance lamps and diffuse reflectance plaques were used for these calibrations, but this technique suffered from variations in the goniometric distribution of irradiance from these lamps, from the diffuser’s spatial transmittance nonuniformity, and from the need to measure the diffuser’s bidirectional reflectance distribution function (BRDF). The goniometric and the BRDF measurement requirement caused propagation of much larger uncertainties. Large-area internally illuminated integrating sphere sources were then developed for calibrating radiometric instruments with a wide field of view. Depending on the construction of the sphere source, the exit aperture of a sphere can provide a uniform (gradients less than 1 %) wide-angle Lambertian source for remote sensing radiometric instruments to view. Space based instrumentation for the ultraviolet (uv) measurement of stratospheric ozone has typically used the former (irradiance lamp/diffuser) calibration method utilizing NIST irradiance lamps and BRDF measurements. Recently the investigators involved in the calibration of a Solar Backscatter Ultraviolet (SBUV-2) instrument approached NIST to characterize the spectral radiance of a large-area internally illuminated integrating sphere source in the wavelength region from 230 nm to 400 nm. This sphere source was subsequently used in a calibration system to intercom-pare a spherical integrator technique with the irradiance lamp/diffuser calibration method. The two techniques gave agreement of approximately 1 % over the range of 250 nm to 340 nm [1].

Recent reports have indicated improvements in the accuracy of derived spectral radiance calibrations based on internally illuminated integrating sphere sources [2, 3]. Previously, the Radiometric Physics Division at NIST has characterized the spectral radiance of integrating sphere sources with exit apertures less than 3 cm in diameter in the Facility for Automated Spectroradiometric Calibrations (FASCAL). An additional instrument has been constructed for specialized radiometric calibrations, primarily radiance mapping of large-area sources and low level spectroradiometry. This mapping spectroradiometer has the capability to map sources with exit aperture sizes up to 60 cm in diameter. The spectroradiometer in this instrument, a single monochromator equipped with a prism predisperser, is primarily optimized in the visible to near infrared (380 nm to 1100 nm), but with appropriate filtering can be used to map sources in the UV. A large-area integrating sphere source (exit aperture ≈20.3 cm) was characterized for NASA’s SBUV-2 program in the wavelength range of 230 nm to 400 nm. Using the mapping spectroradiometer, a high definition map of the spectral radiance of the sphere source was determined at two wavelengths, 300 nm and 400 nm. The two wavelengths were mapped to determine if there are significant spectral gradients, as well as any spatial gradients in the radiance across the exit aperture of the sphere. The final spectral radiance of the sphere source was determined in FASCAL. This paper describes the results of these measurements.

## 2. Material

One internally illuminated integrating sphere source (Unisource 2000), manufactured by Labsphere^1^ was supplied by NASA for these determinations. This sphere source was approximately 50 cm in diameter with a 20.3 cm diameter exit aperture. The internal light sources consisted of two 45 watt tungsten halogen lamps run in series at 6.631 A, direct current and two 150 watt tungsten halogen lamps run in series at 6.022 A, direct current. Lamp currents were set so that both sets of lamps had approximately the same color temperature. Lamp currents were measured by the voltage drop across NIST calibrated shunts. The sphere source was also equipped with a photopic detector and a manufacturer supplied digital photometer. The photopic detector was mounted in the front side of the wall of the sphere looking toward the internal rear wall of the sphere. This provided a means for independently monitoring changes in the output of the integrating sphere source. The photopic detector did not respond in range 230 nm to 400 nm where measurements were made, but it can still be used to indicate if the sphere source output is changing.

## 3. Experimental Procedures

**Fig. 1:**
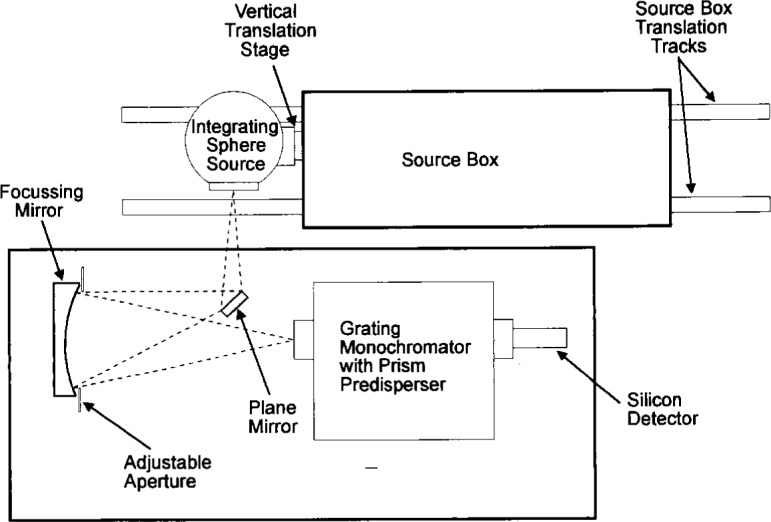
Spectral radiance spatial mapping setup.

The detailed spatial uniformity measurements of the sphere source exit aperture were performed using a mapping spectroradiometer instrument constructed at NIST. This instrument’s foreoptic design is similar to FASCAL but with a significantly lower *f*-number monochromator and imaging system. The instrument performs specialty calibrations using transfer standards from FASCAL. A schematic diagram of the spectral radiance spatial mapping setup is shown in Fig. 1. The monochromator used is a 0.67 m (*f*/4.7) McPherson scanning monochromator equipped with a prism predisperser and a silicon detector. The target area viewed by the monochromator was approximately 0.6 mm wide by 0.8 mm high (the same target area as used in FASCAL) and the bandpass was about 10 nm. The solid angle used to make the measurements, as defined at the center of the target area, had a pyramidal shape but with an approximately square base. The size of the solid angle is defined by the vertex angles at the pyramidal apex, the vertex angle being 12° in the vertical plane and 12° in the horizontal plane. (An aperture in front of the focussing mirror can be adjusted to select smaller input solid angles if they are needed.) All translation stages in the positioning system are computer controlled and servo-motor driven using integral absolute encoders with a linear positioning uncertainty less than 0.01 cm. The integrating sphere source was mounted on a platform attached to a vertical translation stage which provided a 60 cm range of motion and the horizontal motion was provided by translation of the source box. The mapping measurements were made at 1.0 cm increments in the vertical and the horizontal and were referenced to the center position of the exit aperture.

The spectral radiances of the test source from 230 nm to 400 nm were determined in NIST’s FASCAL using the equipment and procedures described in NIST Special Publication 250-1, “Spectral Radiance Calibrations” [4]. The spectral radiance of the test source was determined by comparing its output to the output of a blackbody of known temperature. The blackbody temperature was determined by comparing it to a standard pyrometer lamp at 654.6 nm. The values of spectral radiance measured for the sphere source are associated with a specific target area on the surface plane of the exit aperture. The test source was oriented so that the plane of the exit aperture was normal to the optical axis of the spectroradiometer. The spectral radiance calibration was performed for a rectangular target area 0.6 mm wide by 0.8 mm high located at the center of the sphere source exit aperture. The solid angle used to make the measurements, as defined at the center of the target area, had a pyramidal shape but with a rectangular base. The size of the solid angle is defined by the vertex angles at the pyramidal apex, the vertex angle being 7° in the vertical plane and 3.5° in the horizontal plane. The bandpass of the spectroradiometer for the spectral radiance measurements ranged from about 1 nm at 230 nm to about 2 nm at 400 nm. The source was allowed to stabilize for 30 minutes before measurements were made.

## 4. Results

**Fig. 2:**
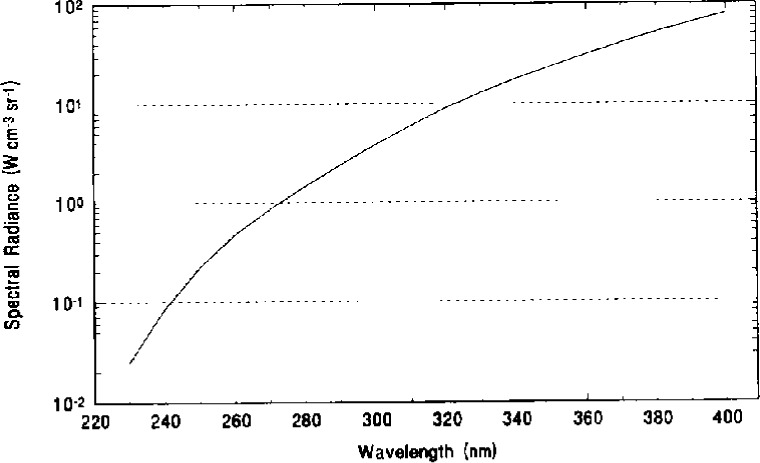
Spectral radiance at the center of the exit aperture of the large-area integrating sphere source.

The spectral radiance of the test source was measured every 10 nm from 230 nm to 400 nm. Figure 2 shows a plot of the measured spectral radiance at the center of the exit aperture of the sphere source. A summary of the expanded uncertainties (2 standard deviation estimates) is shown in Table 1. An explanation of the uncertainty components is given in Ref. [4]. The definitions for all uncertainty estimates cited in this paper are found in Taylor and Kuyatt [5].

**Fig. 3:**
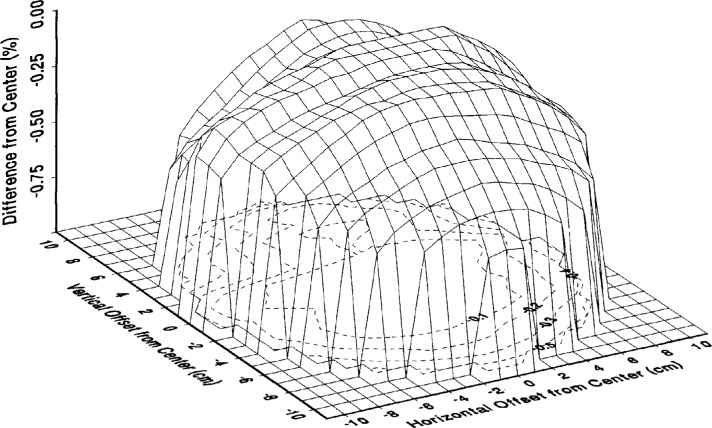
A three-dimensional plot of the spectral radiance mapping of the exit aperture of the large-area integrating sphere source at 400 nm. The spectral radiance percent difference from the center position is plotted. The contour lines are in 0.1 % increments.

Figure 3 is a three-dimensional plot showing the results of spatial mapping measurements performed on the sphere source at 400 nm. Note that the values in this plot are percent differences in spectral radiance from the center position. The mapping was performed relative to the center position of the aperture. The measurement routine consisted of translating the sphere a specified distance in the vertical (in the plane of the aperture) and then translating the sphere in the horizontal (in the plane of the aperture) at a specified increment and making radiance measurements at each horizontal position. Then a new specified vertical position was set and another horizontal scan was performed. This procedure was repeated until the entire aperture was measured. The center position was measured at the start and the end of each horizontal scan and the percent difference of each position was determined by comparing it to the mean of these start and end center measurements. For three mapping measurement scans at 400 nm, the standard deviation was ±0.04 %. Mapping measurements were also made at 300 nm where the signal levels were substantially lower and the data were consequentially noisier. Within the uncertainty of the measurements, the 300 nm spatial mappings agreed with mappings at 400 nm and the ratio of the signals at 300 nm and 400 nm showed no spectral variations of the sphere source.

The results of the mapping measurements can be used to determine the mean spectral radiance and the final radiance uncertainty depending on the size of the target area being viewed by the measuring instrument. A mean radiance correction factor is determined for a given radius of the target area being viewed by calculating the mean of the percent difference from the center of all the measured points circumscribed within the radius and then converting that mean percent difference to a multiplicative factor. The uncertainty of the determination of the mean radiance is dependent on the nonuniformity of the sphere source, the number of of mapping measurements within the area circumscribed by the instrument’s field of view, and the uncertainty of the measurement. The expanded uncertainty of the mean radiance correction factor is determined by calculating the standard deviation of the same data that is used to get the mean of the percent difference from the center and then multiplying it by two. Table 2 shows the calculated mean radiance correction factors and their expanded uncertainties [5] for given radii of target areas being viewed. The small values of the expanded uncertainties are an indication of the good overall uniformity of the spatial mapping. The expanded uncertainty of the spectral radiance for a target area of a given radius is the square root of the quadrature sum of the estimated expanded uncertainty from Table 1 and the expanded uncertainty from Table 2. For this sphere source, which has very good uniformity, the expanded uncertainty of the mean radiance is small and so the expanded uncertainty of the spectral radiance will not change significantly. In other sphere sources where there may be large gradients over the field of view, the expanded uncertainty of the spectral radiance could change significantly.

Care should be taken to perform the spatial mapping using a solid angle that is comparable to the solid angle viewed by the instrument that will be calibrated with the sphere source. A problem could arise, for instance, if the instrument to be calibrated had only a 1° or 2° field of view and the sphere source was mapped using a 12° field of view. The spatial gradients would likely be larger if the sphere source was mapped using an instrument with a 1° or 2° field of view. In order to avoid calibration errors when using the sphere source, the user should determine the field of view of his instrument and request that the sphere source spatial mapping be done using a comparable size field of view.

Calculations were carried out for two specific projected fields of view of NASA’s SBUV-2 instrument on the sphere aperture [1]. For a field of view 13 cm wide by 10 cm high, the mean radiance (with respect to the center position) was −0.085 % with an expanded uncertainty of 0.015 %. For a field of view of 9 cm wide by 6 cm high the mean radiance (with respect to the center position) was −0.028 % with an expanded uncertainty of 0.016 %. Again the expanded uncertainty of the mean radiance is very small and so the expanded uncertainty of the spectral radiance will not change significantly.

## 5. Summary

NIST has the capability to measure the spectral radiance of, and to perform spatial mappings of, large-area integrating sphere sources with exit apertures up to 60 cm in diameter. Commercial large-area sphere sources are available whose spectral radiance can be measured with a 1 % to 2 % expanded uncertainty in the uv and whose spatial mappings are 0.6 % or smaller. The spatial mappings can be used to accurately determine the mean spectral radiance and the expanded uncertainty of the mean spectral radiance for various size fields of view of radiance meters.

## Figures and Tables

**Table 1:** Summary of estimated expanded uncertainties (2 standard deviation estimates) in percent of spectral radiance values. The final expanded uncertainty relative to SI units is the square root of the quadrature sum of the individual uncertainties

Source of uncertainty		Wavelength (nm)				
		230	250	300	350	400
1)	Temperature scale	1.20	1.09	0.92	0.78	0.69
2)	Blackbody quality	0.19	0.16	0.12	0.09	0.06
3)	Calibration of pyrometer lamp relative to the International Temperature Scale (ITS-90)	0.68	0.63	0.52	0.45	0.39
4)	Temperature determination of blackbody and transfer of blackbody spectral radiance to test	1.68	0.53	0.20	0.07	0.07
5)	Short term source drift	0.15	0.15	0.15	0.15	0.15
6)	Polarization effects	0.01	0.01	0.02	0.03	0.04
7)	Size of source effects	0.30	0.30	0.25	0.20	0.15
8)	Lamp current measurement	0.17	0.16	0.14	0.12	0.10
9)	Wavelength measurement	0.24	0.22	0.18	0.16	0.14
Final expanded uncertainty (square root of quadrature sum)		2.23	1.44	1.14	0.96	0.84

**Table 2:** The mean radiance correction factor and the expanded uncertainty (2 standard deviation estimates) of the mean radiance as a function of the radius of target area being viewed. For each radius the mean radiance correction factor and the expanded uncertainty were calculated using the number of circumscribed spatial mapping points from Fig. 3

Radius of target area being viewed (cm)	Number of circumscribed points	Mean radiance correction factor	Expanded uncertainty of the mean radiance correction factor in %
2	13	1.00015	0.027
3	29	1.00004	0.017
4	49	0.99983	0.016
5	81	0.99960	0.014
6	113	0.99942	0.013
7	149	0.99920	0.013
8	197	0.99888	0.014
9	253	0.99853	0.014
10	317	0.99857	0.016

## Footnotes

1 Certain commercial equipment, instruments, or materials are identified in this paper to foster understanding. Such identification does not imply recommendation or endorsement by the National Institute of Standards and Technology, nor does it imply that the materials or equipment identified are necessarily the best available for the purpose.
